# Shape memory polyurethanes crosslinked with castor oil-based multifunctional polyols

**DOI:** 10.1038/s41598-023-42024-1

**Published:** 2023-09-11

**Authors:** Joo Hyung Lee, Seong Hun Kim

**Affiliations:** 1 The Research Institute of Industrial Science, Hanyang University, Seoul, 04763 South Korea; 2 Department of Organic and Nano Engineering, Hanyang University, Seoul, 04763 South Korea

**Keywords:** Chemical engineering, Polymers

## Abstract

As both the industry and academia become more focused on biomass-based smart materials, they are attracting a lot of attention. There has been a significant effort in the field of polyurethane (PU) synthesis to replace polyols used in synthesis with bio-derived polyols. Bio-derived polyols have limited application potential for bio-based PU due to their low functionality. Here, we reported castor oil (CO) based multifunctional polyols prepared by grafting thiols such as 1-mercaptoethanol or α–thioglycerol via a facile thiol-ene click reaction method (coded as COM and COT, respectively). Subsequently, bio-based shape memory polyurethanes (SMPU) crosslinked with prepared polyols were synthesized using a 2-step prepolymer method. By confirming the functionality of the synthesized polyols, it was determined that COT has an OH value of 380 mg KOH/g, which is approximately three times that of CO. The successful synthesis of SMPUs was confirmed through chemical structural analysis. It was also proved that the phase separation between the soft and hard segments was limited due to the increase in crosslinking density. As compared to SMPU crosslinked with CO, the mechanical strength of SMPU crosslinked with COT was improved by 80%, while the elongation was decreased by about 26%. As a result of shape memory behavior analysis, it was confirmed that the outstanding SMPU can be synthesized using CO-based multifunctional polyols.

## Introduction

Shape memory polyurethanes (SMPU) are an important class of polymeric smart materials which can be programmed into temporary shape and returned their original shape by various external stimulus such as heat, light, humidity, pH, microwave, ultrasound or electricity^1–6^. SMPUs are typically synthesized by reacting diisocyanate with a mixture of macroglycol and a low-molecular-weight diol or diamine used as a chain extender. Typical polyol types include polycarbonate polyols, polyether polyols, adipate-based polyester polyols, and caprolactone-based polyester polyols^7–9^. Among these, caprolactone-based polyester polyols exhibit excellent heat resistance, water resistance, and processability and have a wide hardness range; therefore, they are widely used in PU development. The characteristics of SMPU strongly depend on the hydrogen bonding in a PU structure. There are hydrogen bonds within the hard segment and also between the hard and soft segments in PU. Hydrogen bonding within the hard segments occurs between two urethane units, which causes strong phase separation. Hydrogen bonds formed between the hard and soft segments are known to lead to phase mixing. Therefore, the analysis of hydrogen bonding in SMPUs is essential to characterize and design SMPUs^10^.

In recent years, research on PU using bio-based materials has been conducted to replace petrochemical-based chemicals. Oprea synthesized a PU elastomer using polyether-based polyols, hexamethylene diisocyanate (HDI), and a mixture of Castor oil (CO) and 1,4-butanediol as a chain extender and reported that the increased hard segment molar ratio and the presence of a dangling structure formed by introducing CO into the hard segment resulted in an increase in the glass transition temperature (*T*_*g*_) of the PU^11^. Zhang et al.^12^ prepared green polyols by grafting fatty acids extracted from CO into various vegetable oils, such as olive, canola, grape seed, linseed, and CO, which were used to produce PUs, and investigated PU properties. It was found that the higher the hydroxyl value of the modified vegetable oil, the higher the crosslinking density and *T*_*g*_ of a PU.

CO is a vegetable oil extracted from castor beans and is a non-food-based bio-source. CO has attracted attention in various fields of research because of its low cost, abundance, and easy extraction. CO has a triglyceride structure including three fatty acids, and ~ 90% of those fatty acids are composed of ricinoleic acid (12–hydroxy–cis–9–octadecenoic acid) which is an aliphatic unsaturated chain with a double bond at the C-9 position^13^. Because of the advantageous properties of CO, research on utilizing CO for preparing various types of PUs without further modification has been actively conducted for fabricating coating materials, elastomers, foams, and adhesives^14–17^. However, the application of CO-based PUs is limited because of the characteristics such as relatively low mechanical properties and low productivity. To overcome these limitations, the functionality of such PUs has been improved through the modification of CO, such as through epoxidation, transesterification, ozonolysis, and radical addition^18–21^. In previous studies, we reported a PU foam with improved compressive properties when prepared using a polyol that gradually improved the functionality of CO by introducing thiol into CO using a thiol-ene click reaction^22^. We also reported novel bio-PU based polymeric solid–solid phase change materials synthesized using the aforementioned multifunctional polyols^23^. However, few studies have been reported on SMPU using CO-based multifuctional polyols as a crosslinking agent.

In this study, multifunctional polyols were prepared by grafting mercaptoethanol or α-thioglycerol (modified polyols hereafter denoted as denoted as COM or COT, respectively) into CO via a thiol-ene click reaction. The success of the grafting reactions for the as-prepared polyols was confirmed by FT-IR and ^1^H-NMR analyses. After confirming that the hydroxyl value of CO gradually increased from 160 mg KOH/g to 270 mg KOH/g and 380 mg KOH/g after mercaptoethanol or α-thioglycerol were introduced into CO, respectively. PUEs were designed using them as crosslinking agents. Semicrystalline polycaprolactone-diol (PCL-diol) was used for the soft segment, and the hard segment comprised HDI and the as-prepared CO-based multifunctional polyol as a crosslinking agent. For comparison, SMPU using CO as a crosslinking agent was also prepared. The chemical structural properties of the prepared PUs and the change of hydrogen bonding in PU according to the type of crosslinking agent were analyzed by FT-IR spectroscopy. The structural, thermal properties, crystallinity, and thermal stability of the SMPUs were analyzed by Attenuated total reflection-Fourier transform infrared spectroscopy (ATR-FTIR), differential scanning calorimetry (DSC), X-ray diffraction (XRD) analysis, and thermogravimetric analysis (TGA). Finally, the mechanical and shape memory characteristics were analyzed by using a universal testing machine.

## Experimental

### Materials

CO was purchased from Yakuri Pure Chemical Co., Ltd. Mercaptoethanol, α-thioglycerol, 2,2-dimethoxy-2-phenylacetophenone (DMPA), and dibutyltin dilaurate (DBTDL) were purchased from Sigma-Aldrich (USA). Ethyl acetate, *N*,*N*-dimethylformamide (DMF), magnesium sulfate anhydrous (MgSO_4_) and sodium chloride (NaCl) were supplied from Daejung Chemical (Korea). HDI was supplied from Wako chemical. Polycaprolactone-diol, CAPA 2200A (molecular weight 2000) was purchased from Perstorp chemical (Sweden). All reagents were used as received without further purification.

### Synthesis of Castor oil based multifunctional polyols

**Figure 1:**
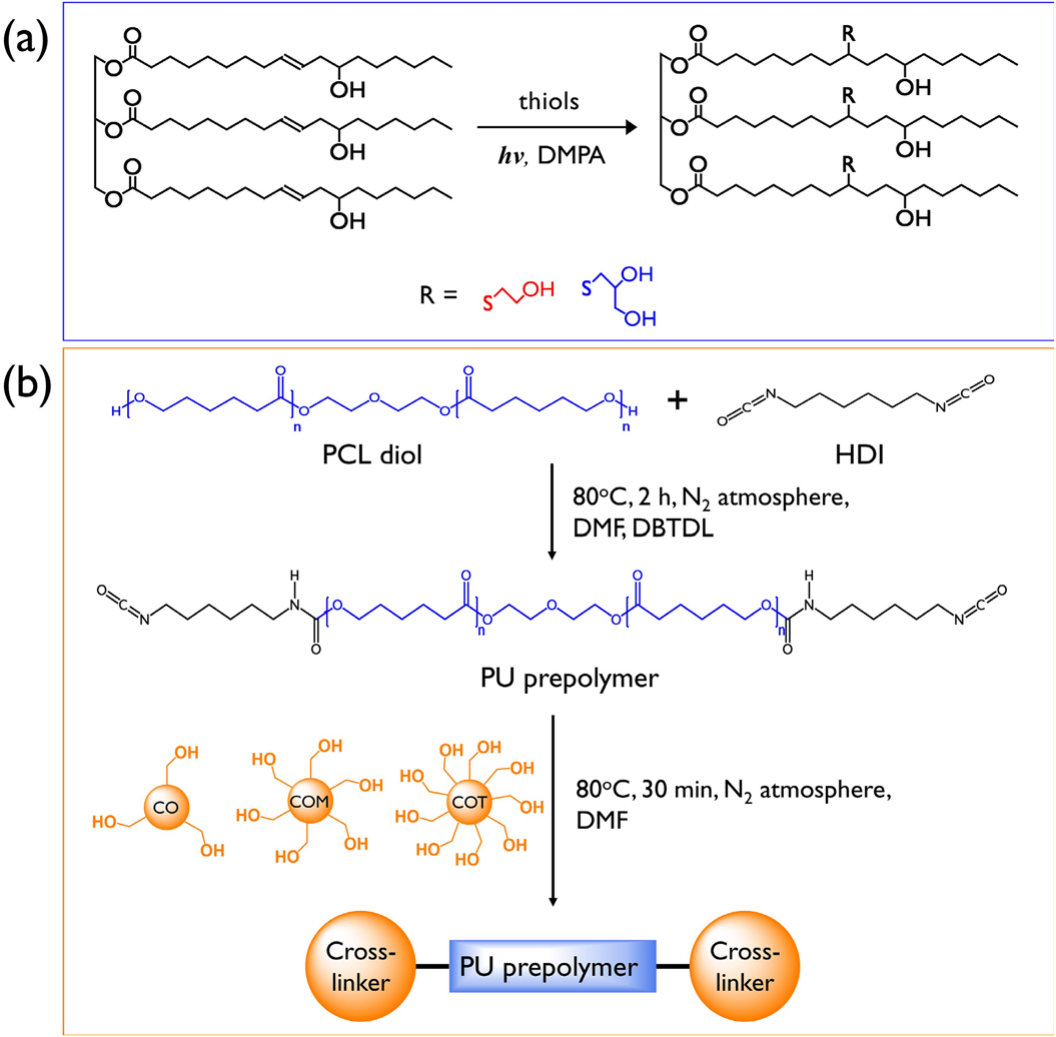
(**a**) Preparation of CO-based multi-functional polyols via the thiol-ene coupling reaction. (**b**) Synthesis pathway of polyurethane elastomer with castor oil based multi-functional crosslinker.

The CO based multifunctional polyols with high functionalities were prepared by our previous method (Fig. 1a)^22–24^. Briefly, the quartz tubes sealed with rubber septa containing the mixture of CO, thiols, photo initiators (DMPA), and solvent (ethyl acetate) were rolled on a tube-roller placed in modified incubator in order to ensure thorough mixing during the reaction. The molar ratio of thiols was set to 4:1 for the C=C double bonds of CO, and DMPA was incorporated as much as 2 wt.% of the entire reactant. After 24 h of the reaction, the products were washed with deionized water and NaCl aqueous for at least five times. The polyols were dried using MgSO_4_ and the solvent was eliminated by rotary evaporation. The final products were dried under vacuum for 24 h prior to use. The polyols were coded as COM and COT treated with mercaptoethanol and 1-thioglycerol respectively. The successful of the COM and COT synthesis was confirmed according to the results of our previous studies^22–24^.

### Preparation of polyurethane

Before preparation of the polyurethane films, the CO, COM and COT were dried at 80 °C for 10 h in vacuo. The polyurethanes were synthesized in a two-step polymerization process as shown in Fig. 1b. In the first step, the NCO-terminated PU prepolymer were obtained by reaction of PCL-diol and HDI with molar ratio of 1:2. The reactants were dissolved in DMF at a concentration of 20 wt.% and stirred at 80 °C for 2 h in a N_2_ atmosphere with a few drops of DBTDL as a catalyst. In the second step, CO, COM, and COT dissolved in DMF were added dropwise and stirred at 80 °C for 3 h. No additional catalyst was added. The solutions were degassed *in vacuo* at room temperature and cast into a PTFE-coated mold. The solvent was allowed to evaporate at 80 °C in a convection oven for 24 h. The final ratios of NCO:OH of the products were 1.05:1. The detailed composition of the PUs obtained are listed in Table 1. The samples were coded as SMPU-CO, SMPU-COM, and SMPU-COT as CO, COM, and COT are used as a crosslinking agent.

**Table 1:** Composition of SMPU-CO, SMPU-COM, and SMPU-COT.

Sample	Functionality ratio of PCL diol/HDI/crosslinker	Type of chain extenders	Total NCO/OH ratio	Hard segment (HS) content^a^	
				Weight (%)	Mol (%)
SMPU–CO	1:2.1:1	CO	1.05	34.9	74.6
SMPU–COM	1:2.1:1	COM	1.05	28.2	71.8
SMPU–COT	1:2.1:1	COT	1.05	24.9	70.8

^a^Hard segment content was calculated using equation below ${\text{HS}}\;{\text{content}} = \frac{{Weight\;or\;moles\;of\left( {HDI + Crosslinker} \right)}}{{Weight\;or\;moles\;of\left( {HDI + Crosslinker + PCL - diol} \right)}} \times 100$.

### Characterization

ATR-FTIR (Nicolet 760 MAGNa-IR spectrometer) was used to determine the structure of the polyurethanes. XRD patterns were scanned in a 2θ range of 5‒70° at scan rate of 3°/min using X-ray diffractometer (Rigaku, SmartLab). TGA for the PUs were carried out from 30 to 800 °C at 20 °C/min under nitrogen atmosphere using PerkinElmer Pyris 1. Thermal behavior of the SMPUs were characterized by DSC (TA Instrument, DSC Q20). All SMPUs were heated from − 80 to 160 °C at 10 °C/min under nitrogen atmosphere and held at 160 °C for 3 min and quenched to − 80 °C, followed by heating again to 160 °C at the same heating rate. The mechanical properties of the SMPUs were measured using an AND MCT-1150 universal testing machine. Testing specimens were prepared in a dimension of 63 × 3 × 0.3 mm^3^ according to the ASTM 638 standard and the crosshead speed was set to 20 mm/min. The mechanical cyclic recovery test was carried out 5 times up to 100% strain. To quantify the mechanical hysteresis (*H*_*M*_) for the prepared SMPUs, according to Fig. S1, *H*_*M*_ were calculated by equation given below from the difference in area under the loading and unloading curves.

1 $$H_{M} = \frac{{\left| {A_{L} - A_{U} } \right|}}{{A_{L} }}$$

Shape-memory test was carried out using Instron 5960 UTM equipped with environmental chamber.

## Result and discussion

### Structural analysis of SMPUs

SMPUs were successfully synthesized by applying PCL-diol (2000 g/mol) as a soft segment and either CO or the as-prepared COM or COT as a crosslinker. As shown in Fig. 1b, the two-step synthesis was carried out by a prepolymer synthesis step and a crosslinking formation step. Three types of transparent and flexible PU samples were obtained.

**Figure 2:**
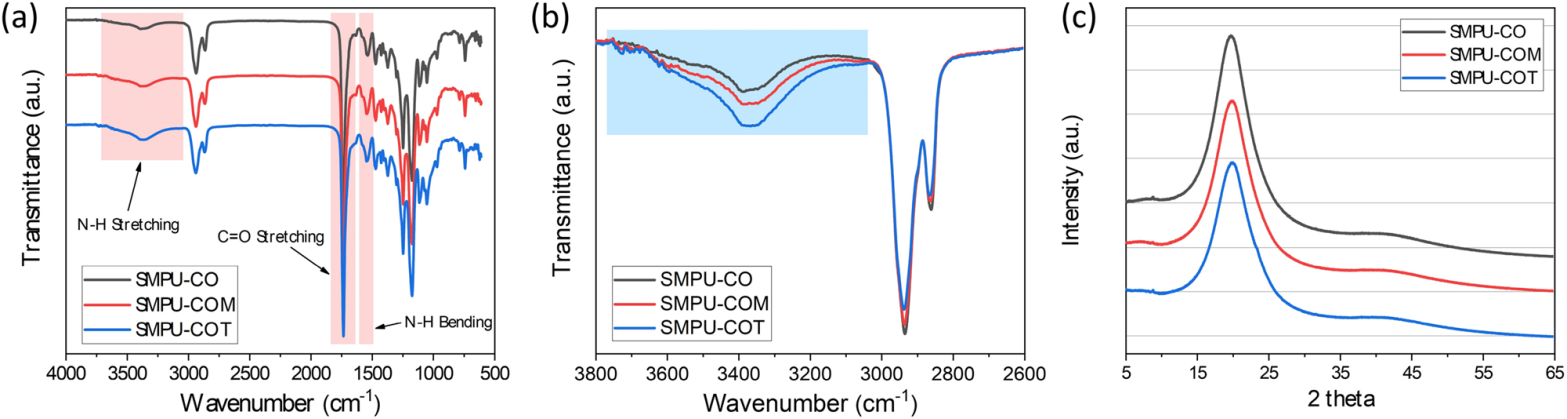
(**a**) Overall FT–IR spectra of the SMPU-CO, SMPU-COM, and SMPU-COT, and (**b**) their amplified > N–H absorption band spectra, (**c**) XRD patterns of SMPU-CO, SMPU-COM, and SMPU-COT.

To analyze the chemical structures of the synthesized SMPUs, FT-IR analysis was performed; the results are shown in Fig. 2. Based on the full FT-IR spectra of the SMPUs (Fig. 2a), the success of the syntheses of SMPU-CO, SMPU-COM, and SMPU-COT was confirmed by the disappearance of the isocyanate peak of HDI, which is known to appear near 2265 cm^−1^. Compared to the PCL-diol spectrum, the spectra of the SMPU samples showed new peaks corresponding to N–H and C=O stretching bands in the urethane bonding at 3380 and 1728 cm^−1^, respectively, and a peak ascribed to N–H bending at 1533 cm^−1^. Moreover, peaks due to the symmetric *sp*^2^ and asymmetric *sp*^3^ stretching bands from the aliphatic chain were observed at 2937 and 2859 cm^−1^, respectively^25^. No significant change in the FT-IR spectra was observed after introducing the crosslinking agents with different OH values into the hard segment. According to several studies, however, we can obtain meaningful information about hydrogen bonding in PUs from the FT-IR spectra^26^. The most widely known approach is to determine the amount of hydrogen bonds by separating the carbonyl peaks. In the PCL-based PU, it is not straightforward to separate the hydrogen-bonded carbonyl peak (about 1700 cm^−1^) and the free carbonyl peak (about 1730 cm^−1^)^27^. In our study, the carbonyl peaks were also overlapped because of the carbonyl peaks of PCL. In such a case, however, the N–H stretching peak can provide meaningful information on hydrogen bonding. It has been found that the sharper the N–H stretching peak, the greater the number of hydrogen bonds; whereas, the broader it is, the fewer hydrogen bonds. The magnified N–H stretching peaks of the SMPU samples are shown in Fig. 2b. The N–H stretching peak was broadened in the order of CO < COM < COT. Based on this, the number of hydrogen bonds in the PU decreased in the order of SMPU-CO > SMPU-COM > SMPU-COT. This phenomenon can be explained by the fact that crosslinking increases when a crosslinking agent with a high functionality is introduced into the hard segment, which interferes with hydrogen bonding between the N–H and C=O groups.

XRD analysis was performed to study the crystallinity of the SMPU samples. The patterns are shown in Fig. 2c, and the pristine PCL-diol pattern is shown in Fig. S2. PCL-diol showed a strong crystal peak at 2*θ* = 21.3°, whereas the XRD patterns of the SMPU samples showed a broad diffraction peak at 2*θ* = 15–30°, which represents the semi-crystalline properties of SMPU; this suggests that the crosslinked bonds were formed between the hard and soft segments after CO, COM, or COT was introduced as a crosslinking agent. This bond formation limited the mobility required for the soft segment to form a crystal structure. In particular, the full width at half maximum (FWHM) of the broad peak was in the order of CO < COM < COT, which confirms that the crosslinking density increased with increasing functionality of the polyols used, resulting in the formation of a more amorphous structure.

### Thermal properties of SMPUs

**Figure 3:**
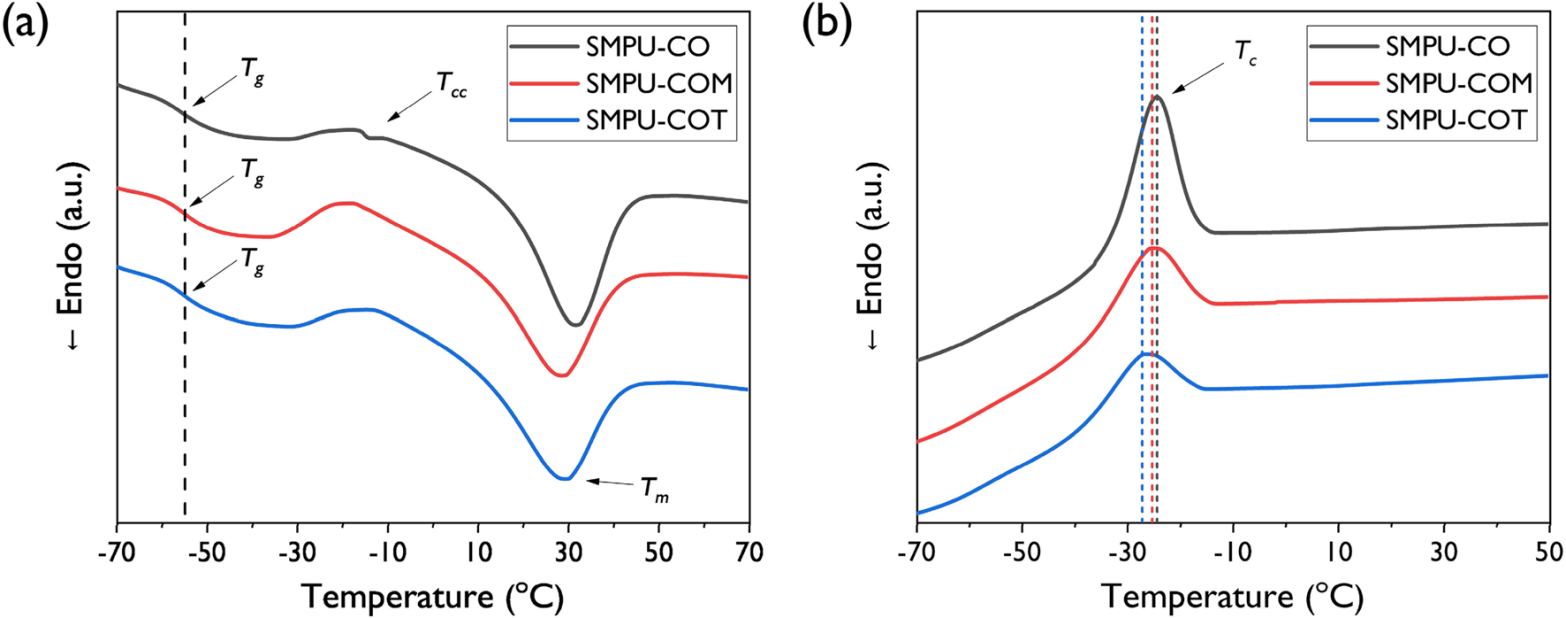
The DSC 2nd heating (**a**) and cooling (**b**) scans of the SMPU-CO, SMPU-COM, and SMPU-COT.

The thermal properties of SMPU-CO, SMPU-COM, and SMPU-COT were analyzed through DSC and the 2nd heating scan are shown in Fig. 3a, and values for melting temperature (*T*_*m*_), transition temperature (*T*_*g*_), degree of crystallinity (*X*_*c*_), and others are listed in Table 2. The peaks for *T*_*g*_, crystallization temperature (*T*_*c*_), and melting temperature (*T*_*m*_) were observed in all SMPU samples. It is known that *T*_*g*_ does not appear in PCL-diol, but *T*_*g*_ became detectable after PCL-diol was incorporated into the crosslinked PU structure. However, almost no change in *T*_*g*_ was observed upon varying the introduced crosslinking agent. It was found in other studies that *T*_*g*_ increased due to inter-domain mixing between the soft and hard segments when small molecule triols such as trimethylolpropane were used as a crosslinking agent. In the cases of CO, COM, and COT, this distinction could not be made because of their very bulky structures. The heat of fusion per gram of PCL-diol in the PU of SMPU-CO, SMPU-COM, and SMPU-COT decreased to 43.9, 34.4, and 32.2 J/g, respectively, as shown in Table 2. The PCL-diol soft-segment crystallinities were 32.4, 25.4, and 23.0%, respectively, which followed the order of SMPU-CO > SMPU-COM > SMPU-COT. Low heat of fusion and low crystallinity indicate the presence of a poorly separated phase. In our study, the crosslinking density increased as the functionality of the crosslinking agent increased, which limited phase separation and thereby decreased crystallinity; this was consistent with the previously reported XRD results. Meanwhile, in addition to glass transition and melting transition, exothermic transition was observed in the heating and cooling scans; this transition is known to be caused by the recrystallization behavior of the PCL soft segment. As shown in Fig. 3b, in the cooling scan, the melt crystallization temperatures were − 24, − 25, and − 27 °C for SMPU-CO, SMPU-COM, and SMPU-COT, respectively; a tendency to gradually shift to a lower temperature was observed. Moreover, the crystal enthalpy was found to gradually decrease to 22.9, 15.3, and 12.7 J/g, respectively, which can be attributed to the disruption of rapid crystal formation upon the increase in the functionality of the crosslinking agent. Meanwhile, in the DSC scans of all SMPUs, the glass transition and melting transition of the hard segment were not observed.

**Table 2:** Thermal properties of SMPU-CO, SMPU-COM, and SMPU-COT.

Sample	*T*_*g*_	*T*_*m*_	*T*_*mc*_	*T*_*cc*_	Δ*H*_*m*_^a^	Δ*H*_*m*_^b^	Δ*H*_*c*_^c^	Δ*H*_*cc*_^d^	*X*_*c*_^e^
	(°C)	(°C)	(°C)	(°C)	(J g^−1^)	(J g^−1^)	(J g^−1^)	(J g^−1^)	(%)
SMPU–CO	− 55	32	− 24	− 17	28.6	43.9	22.9	4.8	32.4
SMPU–COM	− 55	28	− 25	− 18	24.7	34.4	15.3	10.9	25.4
SMPU–COT	− 56	29	− 27	− 13	23.4	31.2	12.7	8.5	23.0

^a^Heat of fusion per gram of the corresponding polymer.

^b^Heat of fusion per gram of PCL-diol in the corresponding polymer (Δ*H*_*m*_^b^ = Δ*H*_*m*_^a^/*ѡ*, where *ѡ* is the weight fraction of PCL-diol in the polymer).

^c^Heat of crystallization per gram of the corresponding polymer in the cooling cycle.

^d^Heat of crystallization (cold crystallization) per gram of the corresponding polymer in the heating cycle.

^e^*X*_*c*_ (soft segment crystallinity) was calculated according to the equation *X*_*c*_ = (Δ*H*_*m*_^b^/Δ*H*_100%_) × 100% = (Δ*H*_*m*_^a^/*ѡ*Δ*H*_100%_) × 100%, where Δ*H*_100%_ is the theoretical heat of fusion of PCL-diol (135.6 J g^−1^) and *ѡ* is the weight fraction of PCL-diol in the polymer. Soft segment crystallinity was determined from the corresponding melting endotherm.

### Thermal stability of SMPUs

**Figure 4:**
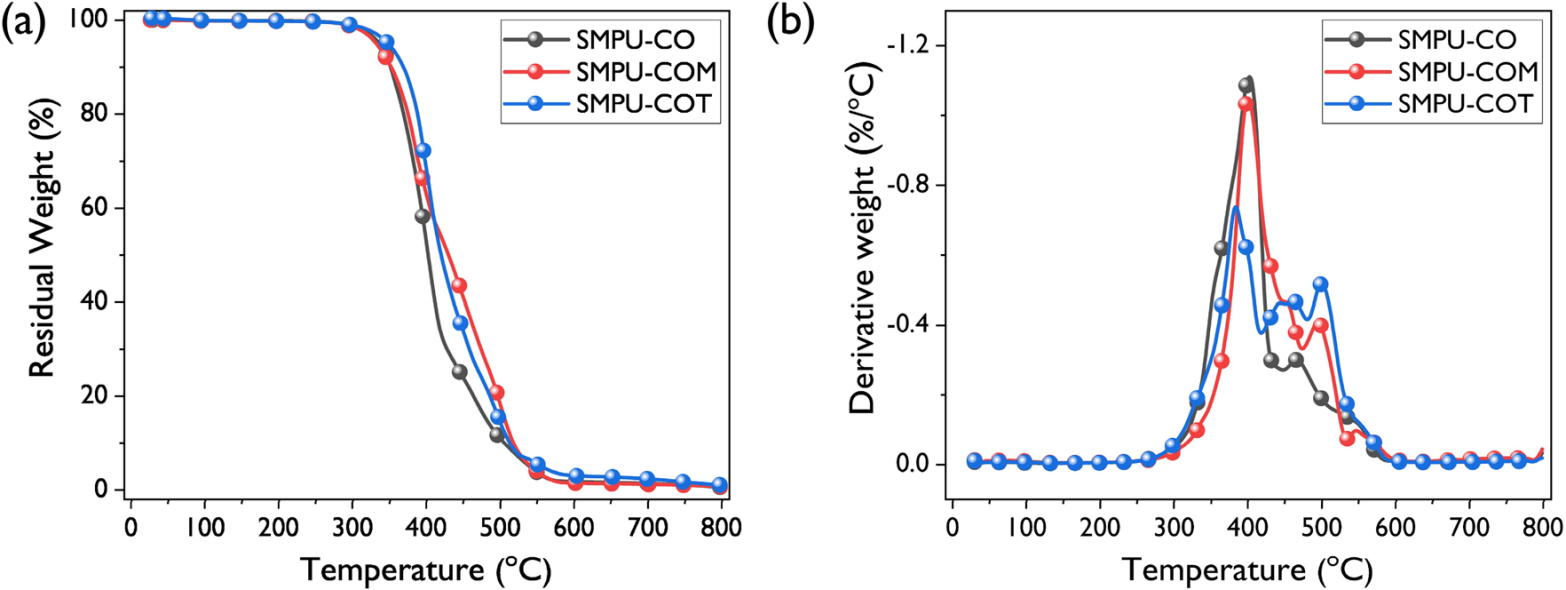
The TGA thermograms (**a**) and derivatives curves (**b**) of SMPU-CO, SMPU-COM, SMPU-COT.

To verify the thermal stability of the synthesized SMPU samples, TGA was performed under a nitrogen atmosphere. TG thermograms and their derivative thermogravimetry (DTG) curves are depicted in Fig. 4, and thermal stability parameters are listed in Table 3. When 5% of SMPU-CO, SMPU-COM, and SMPU-COT decomposed, the temperatures were 332, 336, and 348 °C, respectively, and those for 95% decomposition gradually increased to 538, 540, and 549 °C, respectively. As shown in Fig. 4b, the thermal decomposition process of the SMPU samples proceeded in three stages, which agrees with previous PU research results^28,29^. Table 3 shows the pyrolysis temperatures at each stage measured from the DTG curves. In the first stage, the decomposition of urethane bonds, which impart relatively low thermal stability, began to occur. During the first stage of thermal degradation, SMPU-COT decomposed at the lowest temperature of 385 °C and SMPU-CO at the highest temperature of 403 °C. During the second stage, the oligomerization of the triglyceride structure of the vegetable oil occurred. In the case of SMPU-CO, SMPU-COM, and SMPU-COT, the maximum decomposition temperatures at the second stage were 467, 496, and 501 °C, respectively, which increased significantly with functionality of crosslinkers. This suggested that more thermal energy was required to break the structure of highly branched crosslinking networks. The last stage represents the final decomposition of the remaining materials left after the second stage of thermal degradation; at this stage, SMPU-COT showed the highest decomposition temperature at 562 °C. Based on the TGA results, it was confirmed that the thermal stability of PUs was greatly improved after the introduction of high-functionality crosslinking agents^30,31^.

**Table 3:** Thermal degradation parameters of SMPU-CO, SMPU-COM, and SMPU-COT.

	TGA in N_2_^a^ (°C)			*T*_*dm*_^b^ (°C)		
	*T*_5_	*T*_50_	*T*_95_	1st phase	2nd phase	3rd phase
SMPU–CO	332	403	538	403	467	541
SMPU–COM	336	430	540	401	496	551
SMPU–COT	348	420	549	385	501	562

^a^Thermal degradation temperature at 5, 50, and 95% weight loss.

^b^Temperatures at maximum degradation rates.

### Mechanical properties

**Figure 5:**
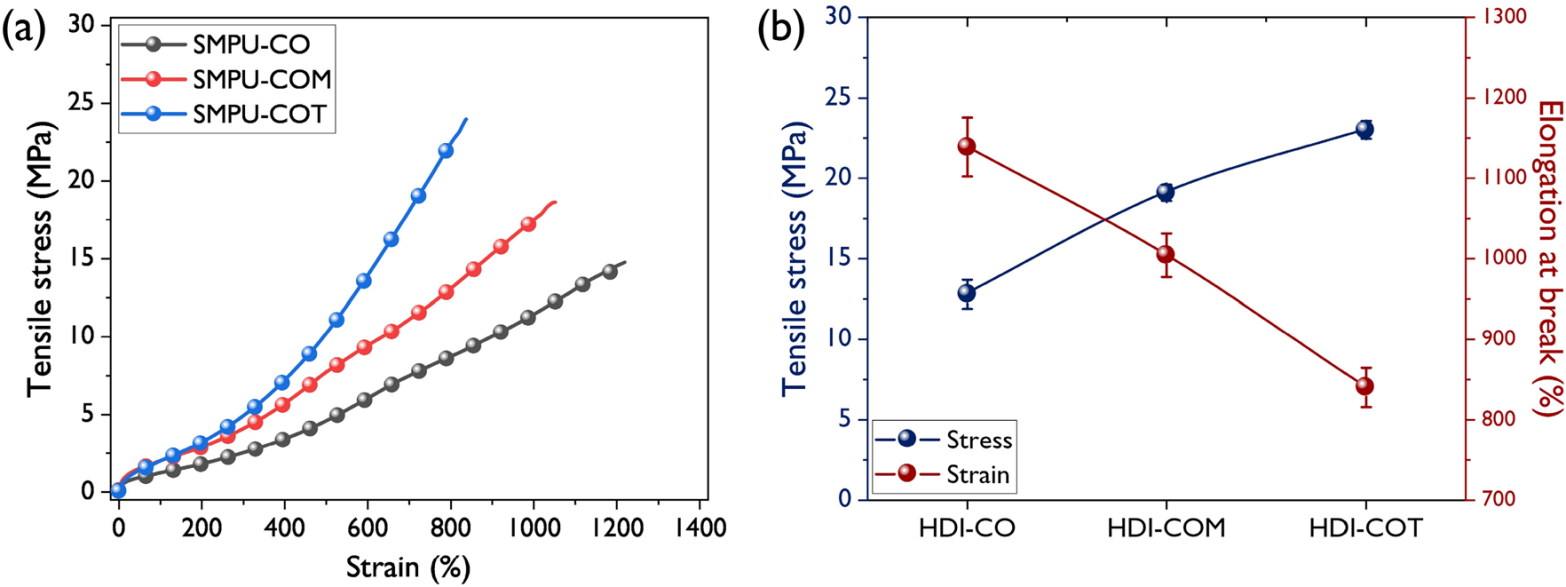
The representative stress–strain profiles (**a**) and changes of mechanical properties (**b**) of SMPU-CO, SMPU-COM, and SMPU-COT.

Figure 5 shows the stress–strain curves and graphs for tensile strength and tensile elongation of the synthesized SMPU samples. All samples had a smooth transition without a yield point in the elastic and plastic deformation regions. As expected, the tensile strength was increased as the crosslinking density increased, but the tensile elongation tended to decrease. SMPU-CO showed a tensile strength of 12.8 MPa and a tensile elongation of 1138%. In comparison, SMPU-COT showed a tensile strength of 23 MPa and a tensile elongation of 840%. The hard segment in the segmented PU acted as a reinforcement. In elastic materials, the tensile strength is inversely proportional to the distance between the crosslinking points^32^. Therefore, the number of crosslinking points increased after the crosslinking agents having high functionalities was introduced, which resulted in a considerable increase in tensile strength.

**Figure 6:**
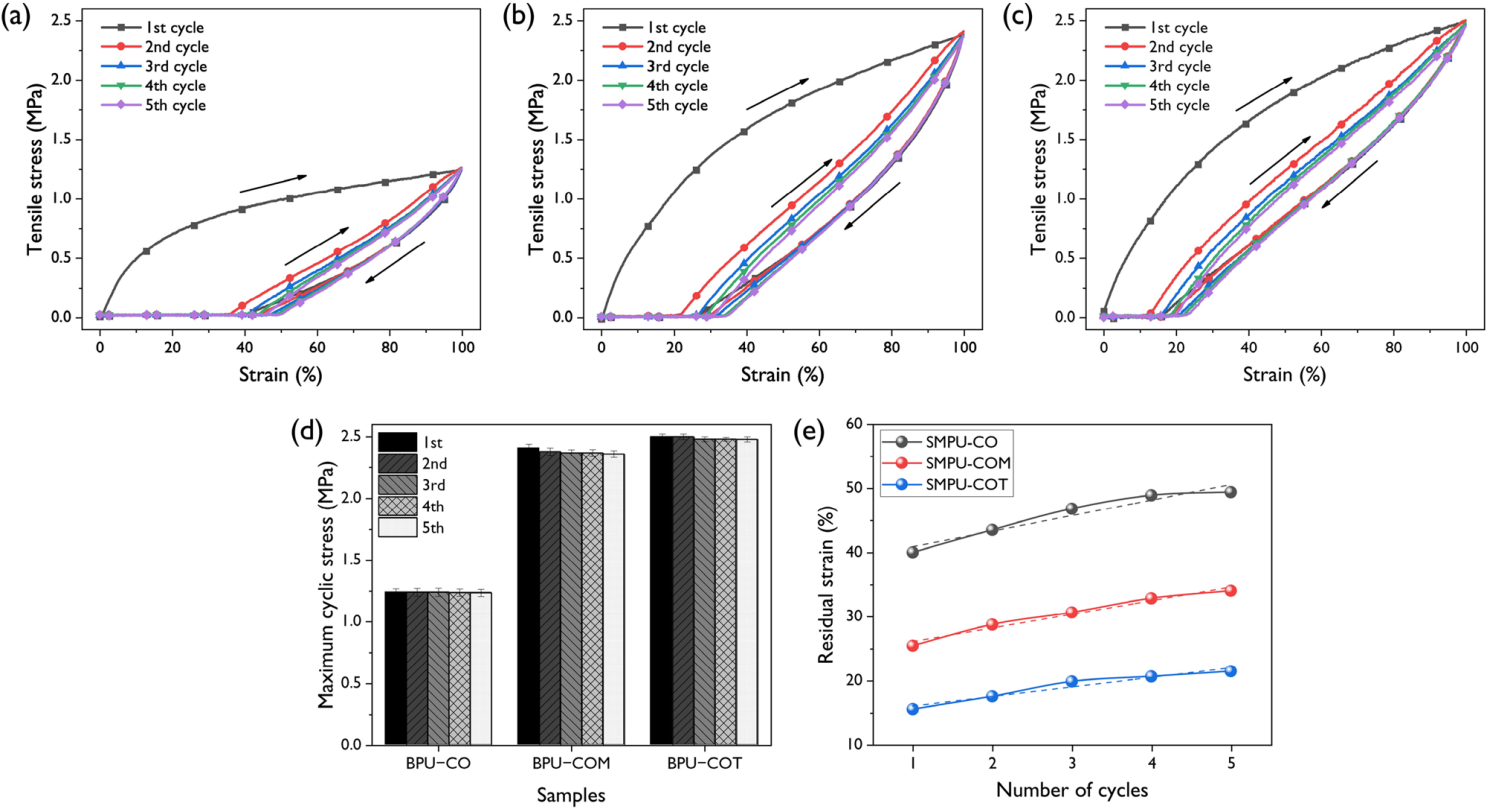
The stress–strain profiles of elastic recoveries at 100% for the (**a**) SMPU-CO, (**b**) SMPU-COM, and (**c**) SMPU-COT under 5 constant cycles at room temperature. (**d**) The changes in maximum cyclic stresses for the SMPUs according to recovery cycles with different crosslinkers. (**e**) Residual strain of the SMPU-CO, SMPU-COM, and SMPU-COT.

To investigate the elastic recovery characteristics of the synthesized SMPUs, five cyclic mechanical tests were performed; the results are shown in Fig. 6. There was a large hysteresis loop between the 1st and 2nd cycles in all SMPU samples. However, no significant difference was observed from the 2nd to 5th cycles. During the 1st cycle, strong softening occurred due to the orientation of the soft segment chain and the rearrangement of the hard segment. This ‘trained’ structure exhibited reversible behavior unless the initial extension was exceeded in subsequent cycles. Figure 6d shows a graph of the change in cyclic stresses of the SMPU samples. In the cases of SMPU-COM and SMPU-COT, the cyclic stress decreased until the 3rd cycle and then remained stable. Strain recovery characteristics are also shown in Fig. 6e. In the 1st cycle, the residual strains of SMPU-CO, SMPU-COM, and SMPU-COT were 40.0, 25.4, and 15.5%, which increased as the crosslinking density increased. Soft segment deformation occurred mainly during the 1st conditioning cycle, which was mostly reversible, while hard segment deformation occurred at the defect in the PU, which was irreversible. Therefore, higher contents of the hard segment and a greater number of crosslinking points led to more prominent elastic recovery characteristics^25^. Moreover, if the initial elongation were not exceeded in subsequent cycles, no further deformation of the hard segment occurred and, thus, high elastic recovery could be maintained.

### Shape memory properties

In order to evaluate the shape memory characteristics, shape memory test using UTM was performed with the following procedures: The sample is first heated above the transition temperature. The original length of the sample is *L*_0_. The melting temperature and crystallization temperature of the soft segment of the prepared SMPUs were considered as the transition temperatures. After sufficient time was given, the sample was stretched to 200% and 400%. The stretched length of sample is n*L*_0_ (n = 2 and 4). Then, the specimen is cooled below its transition temperature, followed by removing tensile load. When the temperature was raised above the transition temperature again, shape recovery of the specimen occurred. A detailed description is shown in Fig. S3. The shape recovered length of sample is *L*_2_. Shape fixity (*R*_*f*_) and shape recovery ratios (*R*_*r*_) are calculated by using follow equations:

2 $$R_{f} = \frac{{\left( {L_{1} - L_{0} } \right)}}{{\left( {2L_{0} - L_{0} } \right)}} = \frac{{\left( {L_{1} - L_{0} } \right)}}{{L_{0} }}$$

3 $$R_{r} = \frac{{\left( {L_{1} - L_{2} } \right)}}{{\left( {L_{1} - L_{0} } \right)}}$$

**Figure 7:**
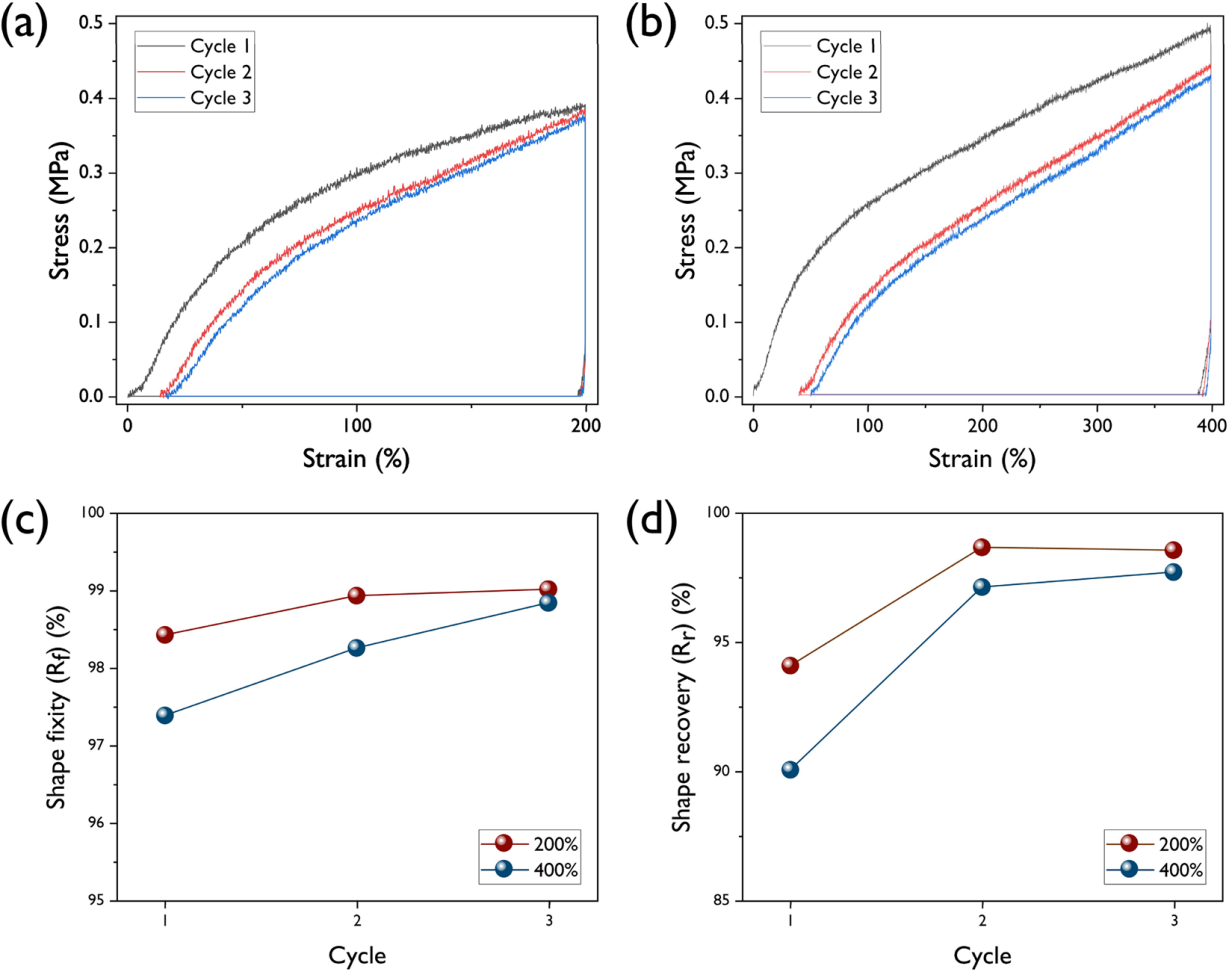
Shape memory behavior of COT at (**a**) 200% and (**b**) 400% strain. (**c**) Shape fixity (*R*_*f*_) and (**d**) shape recovery (*R*_*r*_) values.

**Figure 8:**
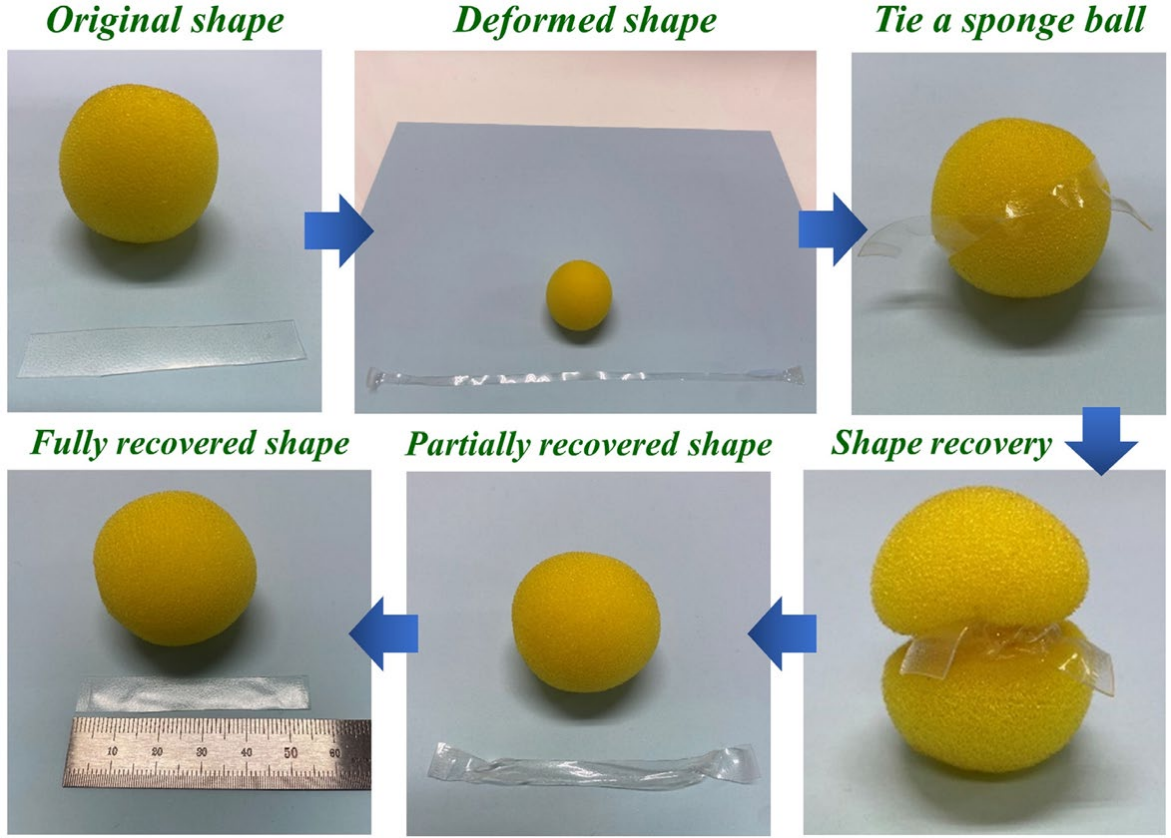
Photographs of SMPU-COT demonstrating the shape memory behavior.

Typical shape-memory testing profile of SMPU-COT at strains of 200 and 400% are shown in Fig. 7a and b and the results are summarized in Fig. 7c and d. The shape-memory tests were carried out in three consecutive cycles. In the case of 200% shape memory test, the shape fixity values were 98.4, 98.9, and 99.0% in the first, second and third cycles, respectively. Generally, shape memory properties are believed to be related to covalent network points in polymers and physical entanglement. It is noted that the hard domains originating from the crosslink of the SMPUs minimize the permanent deformation caused by the creeping of the polymer chain. This is crucial for SMPUs to provide high shape memory retention. It was confirmed that better shape fixity and recovery characteristics were obtained as the functionality of the crosslinker increased. Shape memory characteristics of SMPU-COT was demonstrated and recorded using a digital camera (Fig. 8). First, the SMPU-COT film was deformed in the longitudinal direction at a temperature above the transition temperature and cooled down to fix the deformed shape. A sponge ball was tied once with the modified the SMPU-COT film. When heated to a temperature above the transition temperature again, the SMPU-COT film tightened the sponge ball tightly. The SMPU-COT film was removed from the sponge ball by untying the knot. And heating up to the transition temperature continuously, the shape returned to the original shape.

## Conclusion

In this study, two types of multifunctional polyols having increased hydroxyl values were prepared using a thiol-ene click reaction in CO. Three types of PU were prepared by a two-step synthesis based on the PCL-diol/HDI system by applying either CO or the COM or COT as a crosslinking agent. The successful synthesis of SMPU was confirmed by ATR-FTIR analysis; it was confirmed that hydrogen bonding in the molecule decreased as the functionality of the crosslinking agent increased. Based on XRD and DSC results, it was confirmed that the phase separation between the soft and hard segments was limited because of the increase in the crosslinking density, which decreased crystallinity. TGA results confirmed that SMPU-COT, which decomposed at the highest temperature, exhibited high thermal stability. Based on mechanical property measurements, the tensile strength of SMPU-COT was 23 MPa and the tensile elongation was 840%. Compared to SMPU-CO, the strength was improved by ~ 80%, and the elongation was decreased by about 26%. By introducing a crosslinking agent having a higher functionality into the hard segment, improvement of the elastic recovery property of SMPU was confirmed. As a result of the shape memory behavior analysis, it was confirmed that the higher the functionality of the crosslinking agent introduced into the SMPU led the better shape fixity and recovery characteristics of the SMPU.

### Supplementary Information

Supplementary Information.

## Data Availability

The datasets used and/or analysed during the current study available from the corresponding author on reasonable request.
