# Supplementary material for: Shape memory polyurethanes crosslinked with castor oil-based multifunctional polyols
Source: Sci Rep. 2023 Sep 11;13:14983. doi: 10.1038/s41598-023-42024-1 (PMC10495374; doi:10.1038/s41598-023-42024-1)
Supplement: Supplementary file 1 — Supplementary Information. [file 41598_2023_42024_MOESM1_ESM.docx]

**SUPPORTING INFORMATION**

**Shape memory polyurethanes crosslinked with castor oil-based multifunctional polyols**

Joo Hyung Lee^1,2^ and Seong Hun Kim^1,^ *

*^1^ The Research Institute of Industrial Science, Hanyang University, Seoul, South Korea*

*^2^ Department of Organic and Nano Engineering, Hanyang University, Seoul, South Korea*

*Corresponding Author : Prof. Seong Hun Kim, Ph.D.

E-mail : [kimsh@hanyang.ac.kr](mailto:kimsh@hanyang.ac.kr)


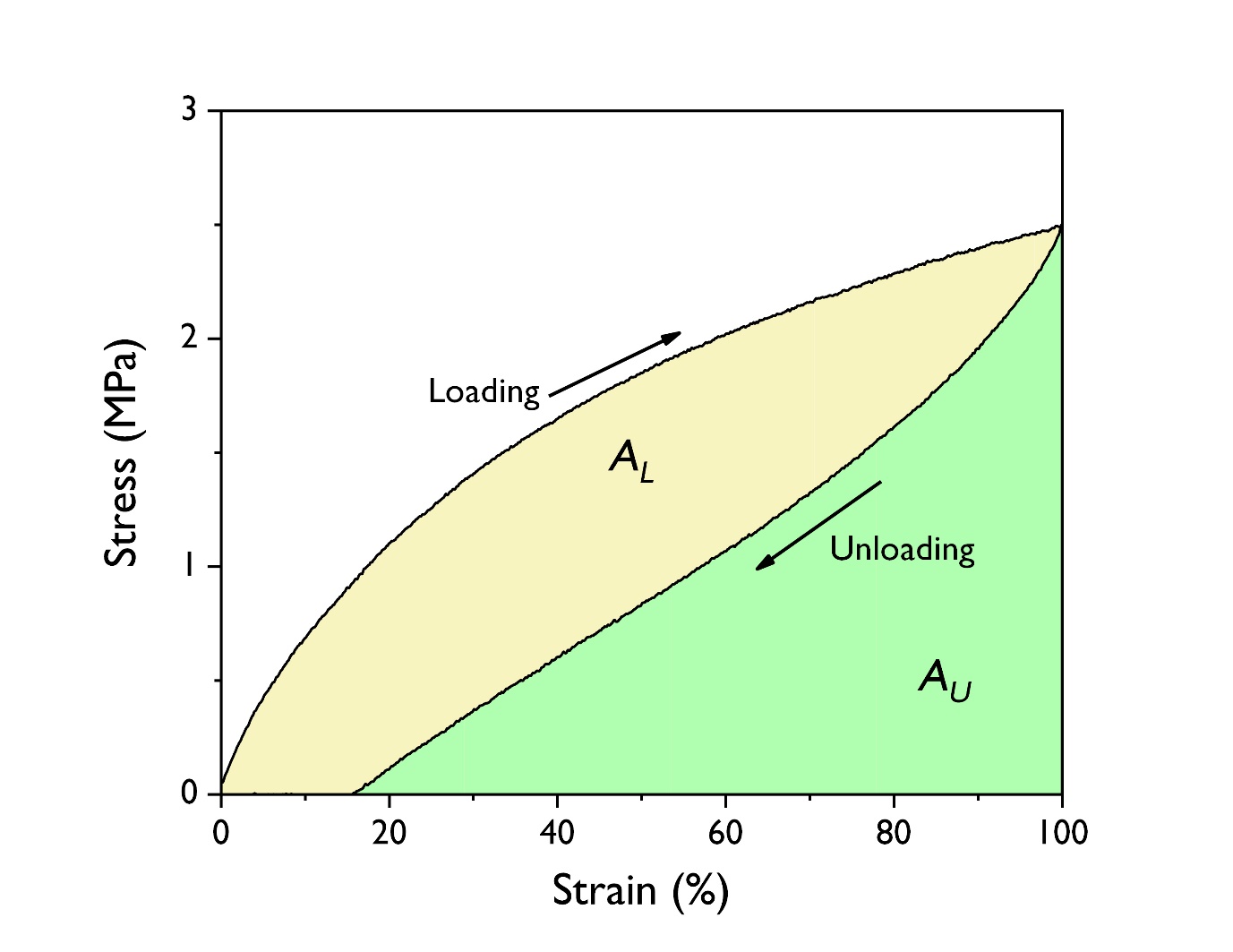


**Figure S1.** Schematic of the mechanical hysteresis quantification using the areas in the stress–strain curve. *A_L_* is the area of the loading curve, and *A_U_* is the area of the unloading curve.


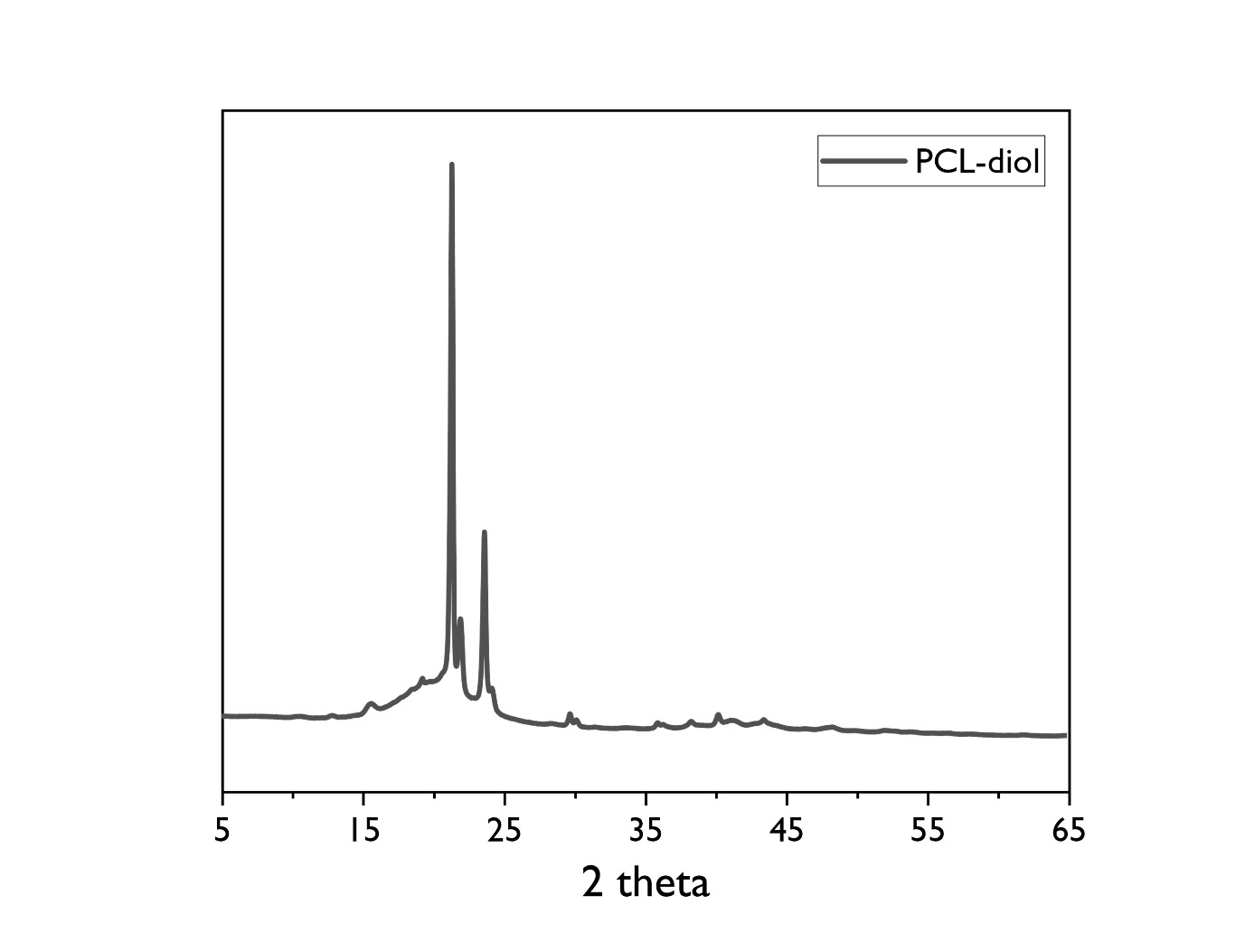


**Figure S2.** X-ray diffraction pattern of PCL-diol.

**
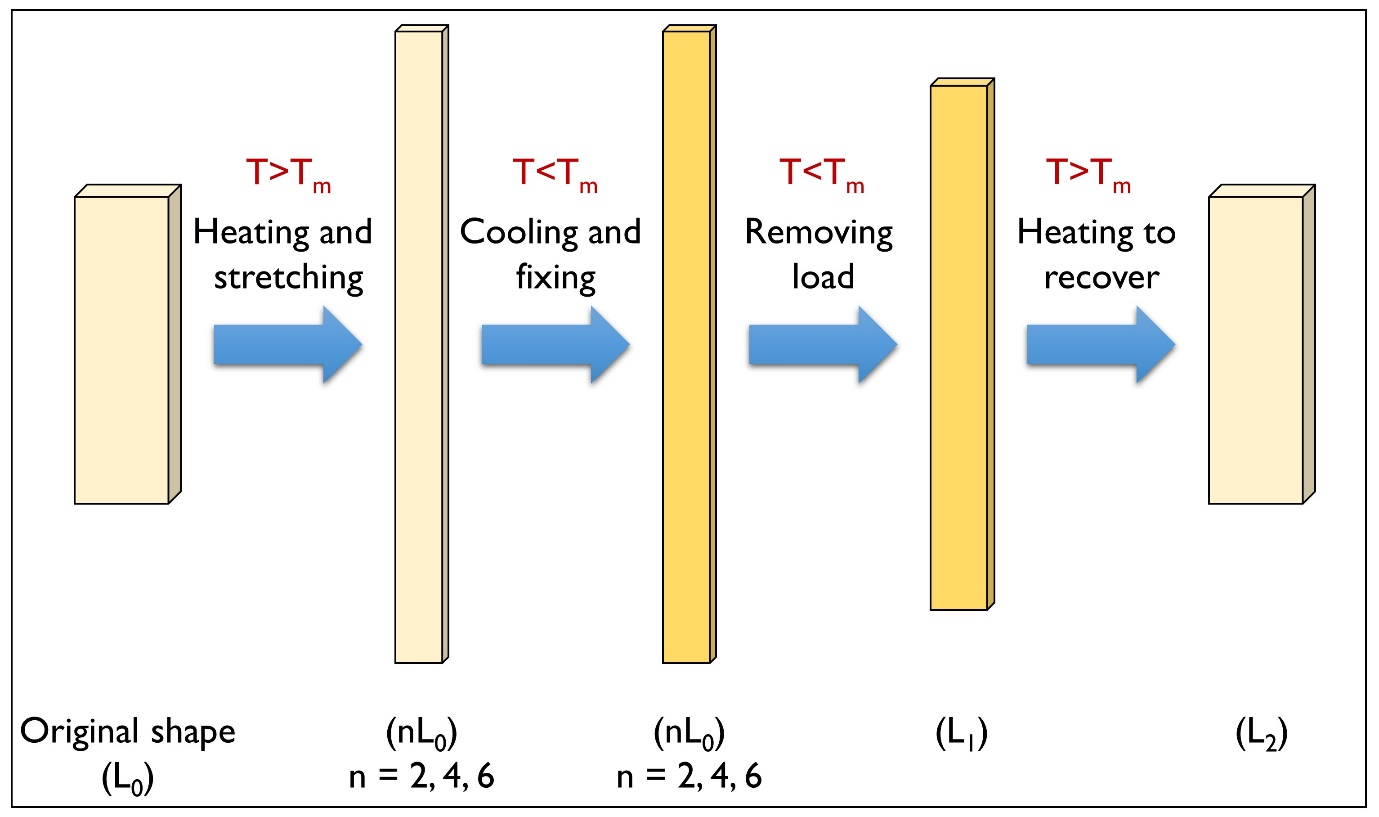
**

**Figure S3.** Schematic representation of SMPU deformation and recovery during shape memory testing.
